# Investigation of Sample Preparation Methods of Dietary Supplements for Total Selenium Determination

**DOI:** 10.1021/acsomega.5c08938

**Published:** 2025-12-04

**Authors:** Larissa M. A. Oliveira, Diogo P. Moraes, Juliana S. F. Pereira

**Affiliations:** Universidade Federal do Rio Grande do Sul, Instituto de Química, Porto Alegre, RS 91501-970, Brazil

## Abstract

This study investigated three sample preparation methods for digestion of dietary supplements prior to selenium (Se) determination by graphite furnace atomic absorption spectrometry (GF AAS), such as conductive heating digestion, microwave-assisted wet digestion (MWAD), and microwave-induced combustion (MIC). In wet digestion methods, variable nitric acid and hydrogen peroxide concentrations were evaluated. For MIC, a diluted nitric acid concentration was also investigated as absorbing solution, while microcrystalline cellulose was employed as a combustion aid. The optimized conditions were: 250 mg of the sample with 5 mol L^–1^ nitric acid and 30% w/w hydrogen peroxide (160 °C, 5 h) for conductive heating; 350 mg with 2.5 mol L^–1^ nitric acid and 30% w/w hydrogen peroxide (180 °C, 40 min) for MWAD; and 200 mg of sample, 300 mg of microcrystalline cellulose, and 0.25 mol L^–1^ nitric acid as the absorbing solution (29.2 bar, > 1000 °C during the combustion reaction and 180 °C, 20 min in the reflux step) for MIC. Digestion efficiency was evaluated by residual carbon content (RCC) and residual acidity (RA) in final solutions. The greenness of each method was evaluated by using the AGREEprep software. Selenium was quantified by GF AAS using Pd as the chemical modifier using pyrolysis and atomization temperatures of 1200 and 2000 °C, respectively. The limits of detection (LOD) obtained for Se were 1.71, 0.84, and 0.08 μg g^–1^ for conductive heating, MWAD, and MIC, respectively, with agreement of 96.7–99.5% for the selenium-enriched yeast certified reference material. Although all methods showed good agreement (ANOVA, 95% confidence level), MIC was the most efficient, providing the lowest RCC (0.1%) and RA (0.5%) values, better LOD, and a greenness score of 0.49.

## Graphical abstract

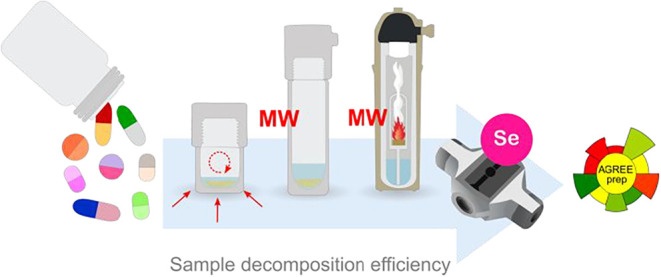

## 1 Introduction

Dietary supplements are over-the-counter products designed to complement the regular diet by providing elevated concentrations of nutrients and bioactive compounds. Common pharmaceutical forms include tablets, capsules, powders, liquids, and chewable gummies.^1^ In dietary supplements containing selenium, the main sources of this micronutrient include inorganic salts (selenite and selenate), used in multivitamin and multimineral formulations, and organic sources such as selenomethionine, probiotic strains such as *Lactobacillus acidophilus*, and selenium-enriched yeast, obtained from the cultivation of *Saccharomyces cerevisiae* in a medium containing sodium selenite.^2–4^

The recommended daily intake (RDI) of selenium varies according to age, sex, and life stage, and also reflects regional differences in the composition of the food chain.^5^ In the United States, the Food and Drug Administration (FDA)^5–7^ recommends an RDI of 55 μg for adults, a tolerable upper intake level (UL) of 400 μg, in Europe, the European Food Safety Authority (EFSA)^8,9^ establishes an RDI of 70 μg for adults and a UL of 255 μg. In Brazil, the Brazilian Health Regulatory Agency (ANVISA)^10^ recommends 34 μg for adults per day, with a maximum limit of 319 μg.

Due to the narrow safety margin for selenium supplementation, even small increases above the recommended concentrations may result in adverse effects.^11^ Furthermore, the lack of strict regulatory requirements for dietary supplements, when compared to pharmaceutical products,^12,13^ raises concerns regarding the quality, storage conditions, stability, and actual selenium content in these products.

Microwave-assisted wet digestion (MWAD) is a widely utilized method for sample preparation in the determination of selenium concentrations, particularly in atomic spectrometry. It offers high efficiency, minimal analyte loss, and a reduced risk of contamination when utilizing closed vessels. Microwave radiation promotes rapid and homogeneous decomposition of complex matrices, enabling digestion pressures up to 100 bar and temperatures ranging from 200 to 300 °C.^14,15^ Nitric acid is one of the most used oxidants in sample digestion due to its strong oxidative capacity, efficiency in organic matrix decomposition, lack of interference with most determinations, and its commercial availability in sufficient purity.^16,17^

In sample digestion, concentrated HNO_3_ is typically employed, resulting in high residual acid concentrations, which can adversely affect the performance of nebulization systems in inductively coupled plasma-based techniques (ICP)^18,19^ or accelerate the degradation of graphite tubes in atomic absorption spectrometry.^20,21^ Despite these advantages, concentrated HNO_3_ is still frequently used, either alone or in combination with auxiliary reagents such as other acids or hydrogen peroxide, for the decomposition of dietary supplement samples.^22–24^ For instance, Hirtz and Günther^22^ proposed Se determination by inductively coupled plasma mass spectrometry (ICP-MS) in 28 dietary supplements after digestion of 500 mg of sample with 6 mL of concentrated HNO_3_ and 2 mL of 30% H_2_O_2_ at 200 °C for 30 min. Similarly, Krawczyk^23^ evaluated MWAD under elevated pressure and temperature conditions for three multivitamin supplements with 4 mL of concentrated HNO_3_, 1.5 mL of concentrated HF, and 1 mL of 30% H_2_O_2_ at 300 W for 20 min before analysis by high-resolution graphite furnace atomic absorption spectrometry (HR-CS GF AAS).

Besides MWAD, other digestion methods can also be employed, ranging from simpler approaches, such as conductive heating digestion, to more advanced systems like microwave-induced combustion (MIC). Augustsson et al.^25^ analyzed Se and other elements in 138 dietary supplements by inductively coupled plasma sector field mass spectrometry (ICP-SFMS). The digestion protocol included overnight predigestion with 10 mL of HNO_3_ and 0.05 mL of HF, both concentrated acids, followed by heating at 110 °C for 120 min. Although effective, the use of HF in wet digestion of dietary supplement samples^23,25^ presents several drawbacks, including incompatibility with a wide range of analytical equipment and formation of insoluble or stable fluoride complexes.

An alternative to using HF for the decomposition of samples containing inorganic matter is MIC. This method is characterized by low reagent consumption, minimal contamination, and enables rapid and complete decomposition of high-mass organic samples.^16,26^ In the MIC process, the pelletized sample is placed on a quartz support along with filter paper and ammonium nitrate, then pressurized with O_2_ and ignited by microwave radiation.^26–28^ For samples with low organic matter content, microcrystalline cellulose can be employed as a combustion aid to promote analyte volatilization.^26,28^ Pereira et al.^29^ employed MIC for the decomposition of multivitamin and mineral supplements using 1.0 mol L^–1^ HNO_3_ and 1.0 mol L^–1^ HCl as absorbing solution for the determination of metals by ion chromatography (IC) while Bitencourt et al.^30^ used 7.0 mol L^–1^ HNO_3_ as the absorbing solution for As determination in dietary supplements samples by ICP-MS. Combustion was performed for 1 min at 900 W. In both works, an additional microwave extraction step was performed to ensure quantitative recovery of the analytes, although the use of cellulose could avoid this additional extraction step.

Previous research has predominantly utilized concentrated nitric acid in MWAD and conductive heating to achieve complete sample decomposition, often followed by dilution before analysis. In this study, diluted HNO_3_ solutions were investigated to reduce the reagent consumption while maintaining the digestion efficiency. Additionally, MIC was introduced as a novel sample preparation approach for the determination of selenium in dietary supplements, which has not yet been explored for this analyte. The three methods: conductive heating, MWAD, and MIC were systematically evaluated in terms of digestion time, efficiency, reagent consumption, and environmental performance using the AGREEprep metric, as well as their applicability for the quantification of selenium by graphite furnace atomic absorption spectrometry (GF AAS). This technique is particularly advantageous for monoelemental analyses due to its high sensitivity and low detection limits, employing pyrolysis conditions between 1000–1200 °C and atomization conditions between 2000–2300 °C, typically with the use of chemical modifiers such as Pd­(NO_3_)_2_ or Pd–Mg­(NO_3_)_2_ in the analysis of dietary supplement samples.^23,24,31–33^

## 2 Materials and Methods

### 2.1 Materials and Reagents

All reagents were of analytical grade. Ultrapure water (resistivity of 18.2 MΩ·cm at 25 °C) obtained from a Milli-Q Integral System (Millipore, USA) was used to prepare all standard solutions and samples. Nitric acid (65% w/w, Sigma-Aldrich, USA) and hydrochloric acid (35% w/w, Merck, Germany) were purified by sub-boiling distillation (Distillacid BSB-939-IR, Berghof, Germany). Hydrogen peroxide (30% w/w, Merck) was used as the oxidizing reagent in the wet digestion methods.

Small disks of ashless filter paper (20 mm diameter, Black Ribbon Ashless, Schleicher and Schuell, Germany) were employed as combustion aids in the MIC method. The disks were first cleaned by immersion in a 10% v/v nitric acid solution for 20 min in an ultrasonic bath (Elmasonic P 60 H, Elma Schmidbauer GmbH, Germany), followed by thorough rinsing with ultrapure water and drying in a laminar flow bench (CSLH-12, Veco, Brazil). Microcrystalline cellulose (Sigma-Aldrich) was also used as a combustion aid, mixed with the sample and then formed into pellets using a pelletizer (13 mm DIE, PerkinElmer) and compressed in a hydraulic press (SSP-10A, Shimadzu). A 6 mol L^–1^ solution of ammonium nitrate (Merck) in water was used as the combustion initiator. Oxygen with 99.99% purity (Air Liquide, Brazil) was used to pressurize the quartz vessels in the MIC system.

Potassium biphthalate (Synth, Brazil) was dissolved in water to prepare calibration curve solutions for residual carbon content (RCC) determination. For residual acidity (RA) determination, a potassium hydroxide solution (Vetec, Brazil) was prepared and subsequently standardized using potassium biphthalate (Synth).

The calibration solution used in the GF AAS was prepared in the range of 20 to 120 μg L^–1^ from the stock solution of 1000 mg L^–1^ Se (Absolute Standard, USA). The chemical modifier was prepared from a 10,000 mg L^–1^ palladium nitrate solution in 15% v/v nitric acid (Merck) to obtain a final solution with a concentration of 1000 mg L^–1^ of Pd­(NO_3_)_2_.

### 2.2 Samples

Four samples of selenium dietary supplements, named A, B, C, and D, were purchased in the local and electronic markets, originating from Brazil and the United States. All of the samples were in tablet form and contained between 30 and 200 μg of selenium per portion. These samples were selected to represent the main categories of selenium-containing dietary supplements currently available, including mineral and multivitamin formulations, probiotic supplements, polysaccharide-based yeast derivatives, and selenium-enriched yeast. The labeled selenium content, tablet mass, origin, and ingredient composition declared on the product labels are summarized in Table S1. For each sample, a representative number of tablets was randomly selected to obtain about 20 g of each sample, ground in an agate mortar and pestle, and homogenized into a fine powder. The homogenized samples were then dried in a forced air oven (400–2ND, Ethik Technology, Brazil) at 80 °C until constant mass, according to procedures previously described.^34^ The dried powders were stored in a desiccator prior to analysis.

For conductive heating and MWAD, the sample masses were introduced directly into the decomposition vessel. For the MIC, the sample was previously homogenized with microcrystalline cellulose prior to pellet formation. The mixture was then compressed using a hydraulic press at 3 tons of pressure for 60 s to produce pellets. Sample A was selected for the optimization of the three decomposition methods evaluated in this work. The selenium-enriched yeast certified reference material SELM-1 (National Research Council of Canada, Canada) was used to evaluate the accuracy of the decomposition methods.

### 2.3 Instrumentation

The sample decomposition was performed using three systems: (i) a conductive heating block digester (TE-007D, Tecnal, Brazil) operating as a closed system, equipped with 12 polytetrafluoroethylene (PTFE) vessels with screw caps and a capacity of 50 mL; (ii) a MWAD system (SpeedWave Four, Berghof, Germany) equipped with closed vessels of modified PTFE (model DAP30), with a maximum capacity of 30 mL; and (iii) a MIC system (Multiwave PRO Sample Preparation System, Anton Paar, Austria) equipped with eight high-pressure quartz vessels (rotor type 8NXQ 80) each having an internal volume of 80 mL. A custom-modified quartz holder (noncommercial model) was employed to position the sample tablets within the quartz vessels during the combustion process.

Total organic carbon (TOC) was measured using an elemental analyzer (PE 2400 Series II CHNS/O, PerkinElmer, USA), while residual carbon content (RCC) was assessed with a carbon analyzer (Multi N/C 2100S, Analytik Jena, Germany) equipped with an AS60 autosampler. The RCC values were expressed as the percentage of organic carbon remaining in the solution after sample digestion.

The RA of the final solutions obtained after decomposition of the samples was determined using an automatic titration system (905 Titrando, Metrohm, Switzerland).

For the determination of the total Se, a graphite furnace atomic absorption spectrometer (PinAAcle 900T, PerkinElmer) equipped with a longitudinal Zeeman background correction system and an autosampler was used. A Se electrodeless discharge lamp (EDL, PerkinElmer) was used as the line source. The analytical signal was measured at 196.03 nm using an integrated absorbance with a spectral slit width of 2.0 nm. Measurements were performed with transversely heated graphite atomizers (THGA) equipped with integrated platform graphite tubes. A sample volume of 20 μL was injected, and the resulting signals were recorded in the integrated absorbance mode. Palladium was used as a chemical modifier, with the addition of 5 μg for each measurement. Argon with a purity of 99.996% (White Martins, Brazil) was used as the protective and purge gas. The optimized parameters for total selenium determination by GF AAS are shown in Table 1.

**Table 1:** Instrumental Parameters for Se Determination by GF AAS

Step	Temperature, °C	Ramp time, s	Hold time, s	Ar flow rate, mL min^–1^
Drying	110	1	30	250
Drying	130	15	30	250
Pyrolysis	1200	10	20	250
Atomization	2000	0	5	0
Cleaning	2300	1	3	250

All statistical evaluations were performed using OriginPro software (Version 2021, OriginLab Corporation, USA), with a confidence level of 95%.

### 2.4 Sample Preparation

#### 2.4.1 Conductive Heating Digestion with Diluted Nitric Acid and Hydrogen Peroxide

Approximately 250 mg of the sample was placed in PTFE digestion vessels. Sample decomposition was performed using variable nitric acid concentrations combined with 2 mL of 30% w/w hydrogen peroxide. For all conditions, the total volume of solution added to the vessels was fixed at 5 mL. The digestion procedure was carried out using a temperature program consisting of four sequential steps: (i) 60 °C for 30 min, (ii) 100 °C for 30 min, (iii) 140 °C for 120 min, and (iv) 160 °C for 120 min. After digestion, the vessels were cooled to room temperature, and the pressure in each container was released. The digested samples were diluted with ultrapure water to a total volume of 25 mL. Then, the vessels were rinsed with water and cleaned with 5 mL of 50% v/v HNO_3_ at 160 °C for 90 min.

#### 2.4.2 Microwave-Assisted Wet Digestion with Diluted Nitric Acid and Hydrogen Peroxide

Approximately 350 mg of each sample was weighed directly into the vessels. The nitric acid concentration at 7, 5, 3.5, 3, and 2.5 mol L^–1^ was initially evaluated in combination with 2 mL of 30% w/w hydrogen peroxide. Subsequently, volumes of H_2_O_2_ of 1, 2, 3, and 4 mL were tested with the previously optimized nitric acid concentration. For all digestion experiments, the total reagent volume in each PTFE vessel was fixed at 8 mL. Microwave digestion was carried out using a temperature program recommended by the instrument manufacturer for the decomposition of multivitamins and multimineral supplements.^35^ The program consisted of: (i) 160 °C, 50 bar, 3 min ramp, 10 min hold, and 1120 W of microwave power (1); (ii) 180 °C, 50 bar, 2 min ramp, 25 min hold, and 1120 W; (iii) 50 °C, 25 bar, 5 min ramp, 15 min hold, and 0 W (cooling step). After digestion, the digested samples were diluted to 25 mL with ultrapure water.

The digestion vessels were cleaned using 8 mL of 50% v/v HNO_3_ and subjected to a microwave heating program as follows: (i) 180 °C, 30 bar, 5 min ramp, 5 min hold, and 1260 W power; (ii) 200 °C, 30 bar, 5 min ramp, 5 min hold, and 1260 W; and (iii) 50 °C, 25 bar, 5 min ramp, 15 min hold, and 0 W (cooling step).

#### 2.4.3 Microwave-Induced Combustion with Diluted Nitric Acid

Sample pellets were prepared without cellulose and with the addition of 100, 300, and 500 mg of microcrystalline cellulose. Each condition was assessed using a filter paper disc previously moistened with 50 μL of 6 mol L^–1^ ammonium nitrate, which was placed in a quartz holder. The holder was then inserted into a quartz vessel containing 6 mL of an absorbing solution (2.5 mol L^–1^ nitric acid). The absorbing solution was evaluated using nitric acid with variable concentrations such as 1.25, 0.50, and or 0.25 mol L^–1^ and using only water. The volume of 6 mL of solution in the vessels was maintained for all tests. After sealing the rotor, the vessels were pressurized with 20 bar of oxygen for 30 s and placed in the microwave oven cavity. The microwave irradiation program used for MIC was: (i) 900 W power for 5 min (ignition and reflux step), and (ii) 0 W for 15 min (cooling step). During combustion, the maximum pressure, pressure rise rate, and temperature were limited to 80 bar, 0.5 bar s^–1^, and 280 °C, respectively. After the vessels were cooled, the internal pressure of the vessels was safely released, and the digests were diluted to a final volume of 25 mL with ultrapure water.

The vessels were cleaned with 6 mL of 50% v/v HNO_3_. The heating program used was: (i) 900 W for 5 min ramp and 10 min hold, and (ii) 0 W for 20 min (cooling stage).

### 2.5 Evaluation of Sample Preparation Methods by AGREEprep

The analytical greenness metrics of the three sample decomposition methods were calculated using the AGREEprep software (version 1.0, Gdańsk University of Technology, Poland)^36^ based on data obtained from the optimized decomposition conditions. In the software, ten criteria related to the main aspects of Green Sample Preparation (GSP) are quantified to calculate the individual scores and the weighted overall greenness index.

## 3 Results and Discussion

### 3.1 Conductive Heating Digestion with Diluted Nitric Acid and Hydrogen Peroxide

For nitric acid concentrations ranging from 7 to 3 mol L^–1^, each combined with 2 mL of 30% w/w hydrogen peroxide, clear digests were obtained only at 7, 6, and 5 mol L^–1^. Under the other conditions, solid residues were observed, indicating incomplete digestion. The RCC and RA of the digests prepared with 7, 6, and 5 mol L^–1^ of nitric acid and 2 mL of 30% w/w H_2_O_2_ are shown in Figure 1. The digestion process was carried out over a heating period of 5 h.

**Figure 1:**
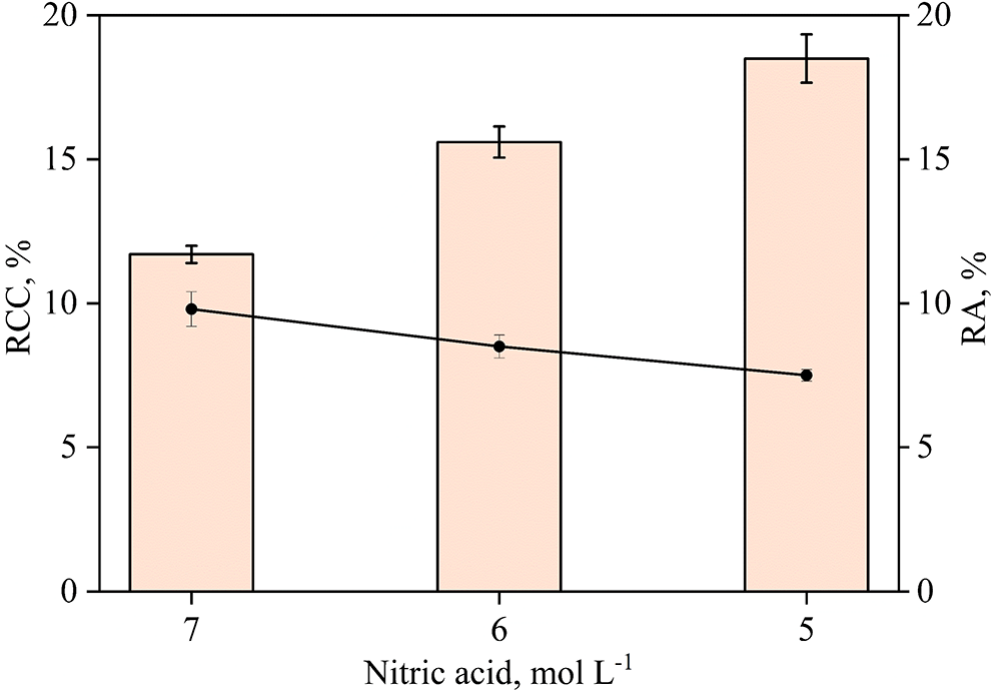
Evaluation of the decomposition of 250 mg of Sample A using a variable concentration of nitric acid combined with 2 mL of 30% w/w hydrogen peroxide by conductive heating. The bars and lines represent RCC and RA, respectively. Results are expressed as mean ± standard deviation for *n* = 3.

Figure 1 shows, as expected, an inverse relationship between nitric acid concentration and RCC. With 7 mol L^–1^ nitric acid, the digest exhibited 11.7% RCC, while lower concentrations resulted in RCC values exceeding 15%. Conversely, RA decreased from 9.8 to 7.5% at the lowest nitric acid concentration. Based on these results, the condition of 5 mol L^–1^ nitric acid combined with 2 mL of 30% w/w hydrogen peroxide was selected due to its lower residual acidity. RA and RCC were 7.5 and 18.5%, respectively.

Although the selected condition presented a higher RCC value, this parameter is not crucial for GF AAS determinations since the pyrolysis step removes the residual carbon before atomization. In contrast, RA has a greater impact on measurement quality, as high acid concentrations can shorten the lifetime of graphite tubes,^20,21^ and affect the sensitivity of selenium quantification.

### 3.2 Microwave-Assisted Wet Digestion with Diluted Nitric Acid and Hydrogen Peroxide

To reduce the residual acidity of the digests and, consequently, the decomposition time, the use of MWAD was evaluated. For this, 350 mg of sample were tested with nitric acid concentrations of 7, 5, 3.5, 3, and 2.5 mol L^–1^, each of them combined with 2 mL of hydrogen peroxide (30% w/w). RA and RCC were determined in the final digestion, and the results are shown in Figure 2.

**Figure 2:**
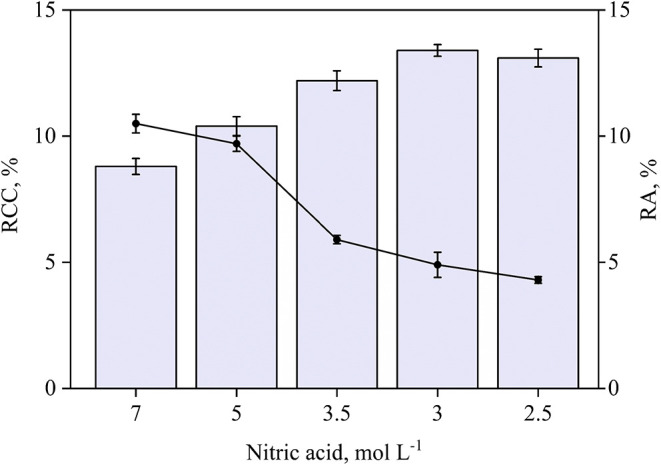
Evaluation of the decomposition of 350 mg of Sample A by MWAD using different concentrations of nitric acid combined with 2 mL of 30% (w/w) hydrogen peroxide. The bars and lines represent RCC and RA, respectively. Results are expressed as mean ± standard deviation for *n* = 3.

Under all conditions of nitric acid concentration, the digests showed no remaining solids. The lowest RCC values were obtained with 7 and 5 mol L^–1^ nitric acid, but with RA higher than 9%. When the acid concentration was reduced, decomposition was efficient, although with slightly higher RCC values, accompanied by a reduction in the RA. Since no significant difference was observed in RCC between 3.5, 3, and 2.5 mol L^–1^ of HNO_3_ (95% confidence level, ANOVA), 2.5 mol L^–1^ nitric acid was chosen for subsequent assays due to its lower residual acidity.

To evaluate the influence of hydrogen peroxide (30% w/w) in the decomposition using 2.5 mol L^–1^ of nitric acid, different volumes were tested, and the results are presented in Figure 3.

**Figure 3:**
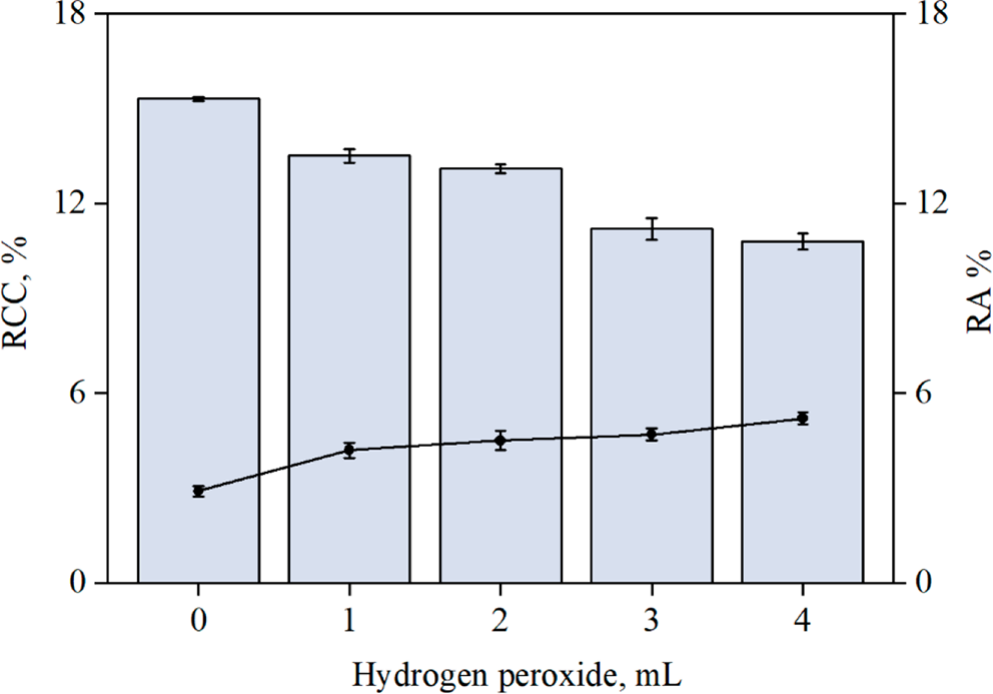
Effect of 30% w/w hydrogen peroxide volume using 2.5 mol L^–1^ nitric acid in the decomposition of 350 mg of Sample A by MWAD. The bars and lines represent RCC and RA, respectively. Results are expressed as mean ± standard deviation for *n* = 3.

In the absence of hydrogen peroxide, the highest RCC was observed, indicating a low decomposition efficiency. When 1 to 4 mL of hydrogen peroxide was added, RCC values decreased to below 14%, and RA ranged from 4.2 to 5.2%, reflecting improved decomposition efficiency and nitric acid regeneration under closed-vessel conditions. The lowest RCC and RA values were obtained with 3 and 4 mL of hydrogen peroxide, without a significant difference between them (95% confidence level, *t* test). Therefore, 3 mL of hydrogen peroxide was selected for subsequent experiments due to its lower residual acidity. The RA and RCC values were 4.7 and 11.2%, respectively. This condition was considered suitable for GF AAS determinations as the pyrolysis step effectively removes most of the residual carbon. Furthermore, even with the use of diluted acid and a relatively high sample mass, the MWAD method resulted in a 50% reduction in nitric acid consumption and demonstrated improved decomposition efficiency when compared with conductive heating.

### 3.3 Microwave-Induced Combustion with Dilute Nitric Acid

Considering the need to efficiently digest dietary supplements while obtaining digests with low residual acidity and extending the lifetime of graphite tubes for selenium quantification by GF AAS, MIC was selected for this study due to its advantages over conventional wet digestion methods. Its main benefits include the use of diluted solutions to retain analytes, low risk of contamination, faster and efficient decomposition of both organic and inorganic matrices, and the possibility of refluxing after combustion to ensure complete removal of analytes.^26,37^

The method was carried out following previously established and published parameters, including: an initial oxygen pressure of 20 bar; 6 mL of absorbing solution, which is the minimum volume recommended by the equipment manufacturer; 50 μL of ammonium nitrate solution as the combustion initiator; an irradiation time of 5 min; and the use of filter paper disks for positioning the sample pellets on the quartz holder.^27,28,28,37^

Due to the predominantly inorganic composition of the dietary supplements analyzed in this study (approximately 60–70%), the use of microcrystalline cellulose as a combustion aid was evaluated. Previous studies have reported cellulose as an auxiliary agent in the volatilization of metals during the decomposition of inorganic matrices, leading to an increase in the internal temperature of the digestion vessel and consequently facilitating the volatilization of analytes present in the nonorganic fraction.^26,28^

To determine the ideal mass of microcrystalline cellulose as a combustion aid, the effect of its addition in different proportions (0, 100, 300, and 500 mg) on total selenium determination of the final solution was investigated (Figure 4). The method was evaluated for the decomposition of 200 mg of Sample A, using 2.5 mol L^–1^ nitric acid as the absorbing solution, based on the optimized conditions previously established in the MWAD experiments.

**Figure 4:**
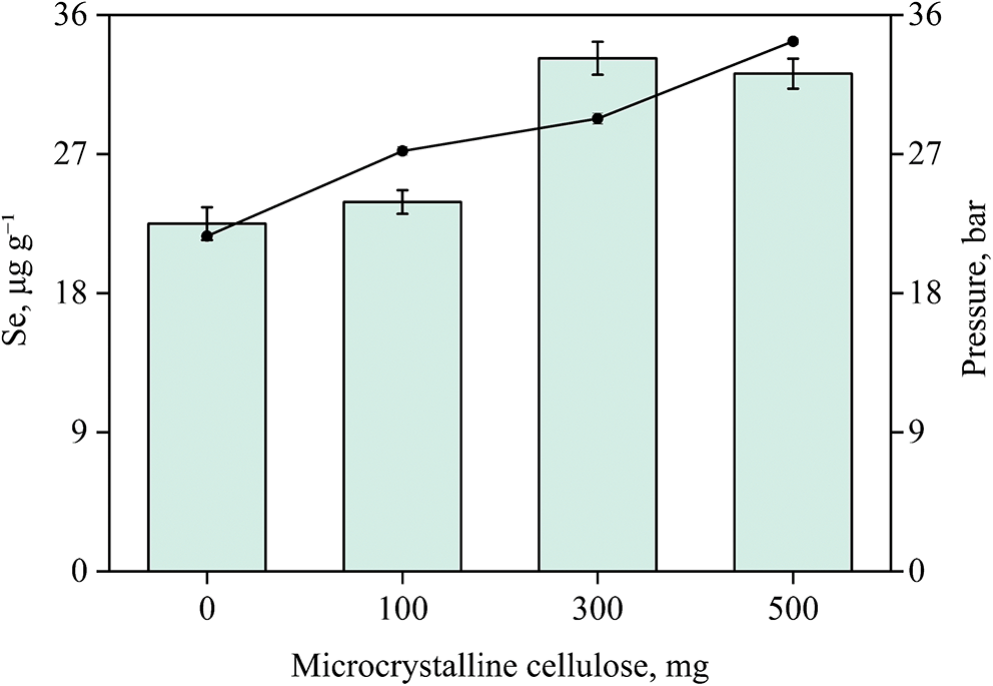
Evaluation of different masses of microcrystalline cellulose for the decomposition of 200 mg of Sample A, considering the maximum pressure reached during combustion. Bars and lines represent the concentration of selenium recovered after MIC and the pressure reached in the system, respectively. Results are expressed as mean ± standard deviation for *n* = 3.

After decomposition, all absorbing solutions obtained under the tested conditions appeared clear, although solid residues were observed at the bottom of the quartz holder. These residues were attributed to the inorganic fraction of the sample and did not affect the analytical performance of the method.

The internal pressure values recorded during the experiments are listed in Figure 4. The maximum pressure ranged from 21.7 to 34.2 bar, remaining below the critical threshold of 40 bar, which is considered safe for the experimental system. The selection of the most suitable condition was based on the total selenium content determined in Sample A.

In the absence of cellulose, or with only 100 mg added, selenium concentrations in the final solutions were below 25 μg g^–1^, approximately 17% lower than the reference value reported on the sample label (30 μg g^–1^). This observation may be attributed to the partial removal of the element in the inorganic fraction of the sample, as indicated by the relatively low pressure reached during the sample combustion.

The addition of 300 and 500 mg of cellulose resulted in internal pressures of 29.2 and 34.2 bar, respectively, and selenium concentrations of 33.12 and 32.21 μg g^–1^, respectively, with no significant difference (95% confidence level, *t* test). Therefore, 200 mg of Sample A combined with 300 mg of microcrystalline cellulose was selected as the optimal condition for subsequent MIC experiments. Larger sample masses were not investigated to avoid exceeding the safe 2:3 sample to cellulose ratio, which could compromise the safety limits of the equipment and operator.

To reduce the RA of the digests,1.25, 0.50, and 0.25 mol L^–1^ nitric acid and water were used as the absorbing solution (Figure 5). The experiments were conducted using 200 mg of the sample and 300 mg of microcrystalline cellulose, previously established as optimal conditions.

**Figure 5:**
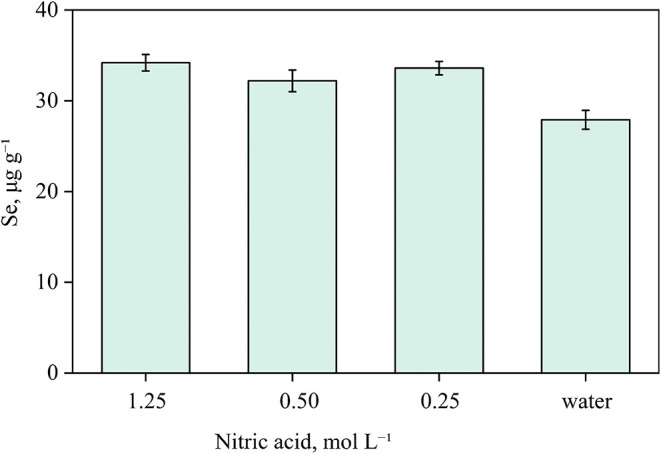
Evaluation of different concentrations of nitric acid and water as absorbing solutions for the decomposition of 200 mg of Sample A and 300 mg of microcrystalline cellulose. Bars represent the concentration of selenium recovered after MIC. Results are expressed as mean ± standard deviation for *n* = 3.

The use of water resulted in the lowest selenium values (27.92 μg g^–1^), while the diluted nitric acid solutions yielded higher concentrations: 33.28, 32.25, and 32.61 μg g^–1^ for 1.25, 0.50, and 0.25 mol L^–1^, respectively. These values showed no statistical difference (95% confidence level, ANOVA). Therefore, the lowest HNO_3_ concentration, 0.25 mol L^–1^, was selected as the absorbing solution for the quadruplicate sample decompositions. Under these conditions, the digest presented an RA of 0.5% and an RCC of 0.1%.

### 3.4 Optimization of Total Selenium Quantification Method by GF AAS

Initially, pyrolysis and atomization curves were investigated to define the optimal temperatures for total selenium quantification by GF AAS. The chemical modifier of 5 μg of Pd was employed, using a selenium standard solution (50 μg L^–1^) and a digest of Sample A prepared by MWAD, diluted 5 times.

The pyrolysis curve was performed by varying the temperature between 900 and 1600 °C, while the atomization temperature was kept constant at 1900 °C. Subsequently, the atomization temperature was adjusted between 1800 and 2300 °C, using the previously selected pyrolysis temperature. The results obtained are presented in Figure 6.

**Figure 6:**
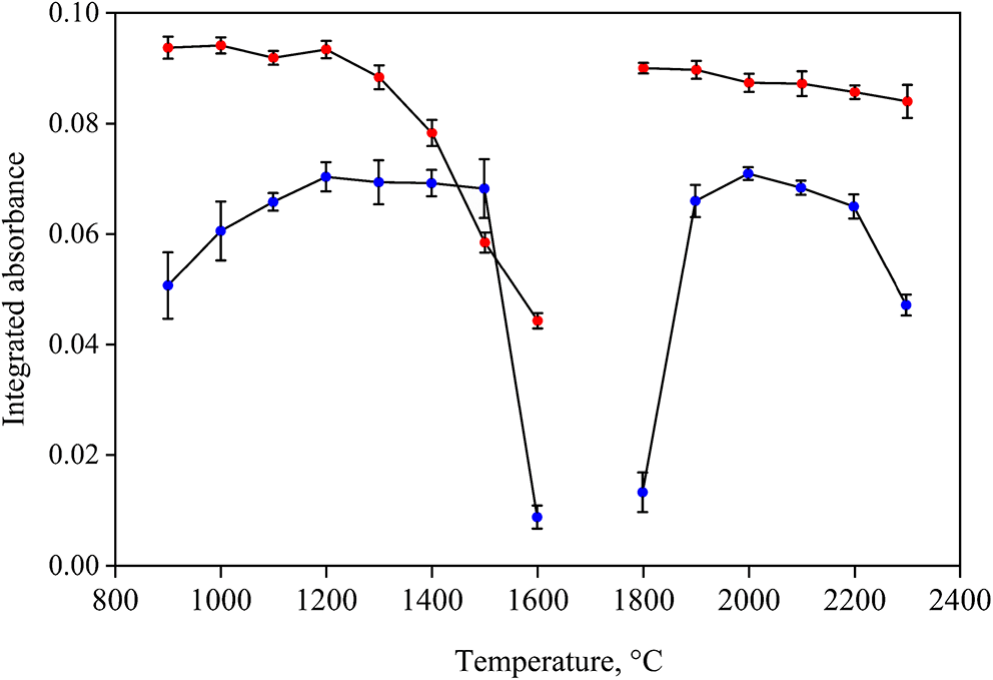
Pyrolysis and atomization temperature curves for Se determination by GF AAS, employing 15 μL of 50 μg L^–1^ selenium standard solution (red ●) and digests of Sample A obtained after decomposition by MWAD (blue ●), both in the presence of 5 μL of Pd. Vertical bars indicate the standard deviation for *n* = 3.

As shown in Figure 6, for the standard solution, the analyte exhibited a stable absorbance between 900 and 1200 °C, with no significant differences at the 95% confidence level (ANOVA). Above 1300 °C, a decrease in signal occurred due to analyte volatilization. In contrast, the digestates showed consistent absorbance between 1100 and 1500 °C, but a decrease below 1100 °C, which can be attributed to the presence of concomitants in the matrix that were not fully volatilized, effectively trapping the selenium species and preventing their release into the gas phase. As the temperature increased, these concomitants were volatilized, allowing the analyte to be released and atomized. This effect may have shifted the optimal temperature range to higher values in the digestates compared to the aqueous medium.^38–40^ Considering these results, 1200 °C was selected as the pyrolysis temperature, as it represents a compromise condition and is widely reported in the literature for selenium determination in supplements and similar matrices.^33,41^

For atomization, absorbance signals were similar between 1900 and 2100 °C for both the standard and digested solutions, with no statistical difference (95% confidence level, ANOVA). Therefore, 2000 °C was selected as the optimal atomization temperature.

Regarding the thermal behavior of the analyte in the digests obtained by the three decomposition methods, it was observed that the digestates from conductive heating and MWAD exhibited increased background signals and broader peak bases compared with those obtained by MIC, as shown in Figure S1. These effects can be attributed to the specific characteristics of the digestates produced by each decomposition method. In wet digestion methods, inorganic components remain in solution as soluble salts, such as sulfates, chlorides, and nitrates, depending on the reagents employed.^16^ As summarized in Table S1, Sample A contained several additional elements besides selenium, which may contribute to matrix-related interferences during atomization. The presence of other metals in solution can influence selenium volatilization, even in the presence of the chemical modifier Pd, resulting in signal broadening and increased residence time of the analyte within the graphite tube.^42^ Furthermore, NO molecules originate from the decomposition of HNO_3_ used during sample preparation and can also contribute to spectral interference.^43^ This type of interference tends to be more pronounced in methods employing higher concentrations of nitric acid, such as conductive heating and MWAD. In contrast, the MIC method utilized absorbing solutions with low nitric acid concentrations, resulting in digestates with lower RA values compared to the other methods. This residual acidity may have contributed to mitigating NO-related interference during spectrometric measurements.

To confirm the influence of nitric acid concentration on the background signal of digests obtained by wet digestion methods, the solutions obtained from conductive heating and MWAD were diluted 7.5 and 5 times, respectively. Then, different nitric acid concentrations were added to simulate the conditions of analyzing the digests in different dilutions or without further dilution. For this purpose, nitric acid concentrations of 1.0, 2.5, 5 and 7.5% were evaluated in conductive heating digests, and 1.0, 2.5, and 5% were evaluated in MWAD digests. The results are shown in Figure 7.

**Figure 7:**
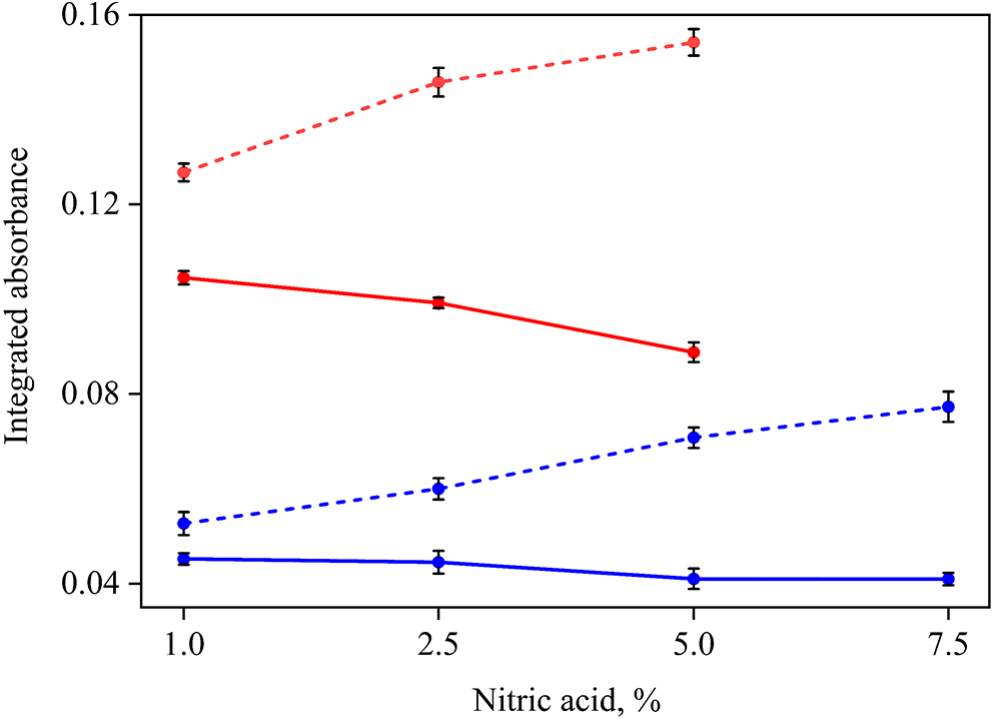
Influence of nitric acid concentration on the analyte (―) and background (---) signals in Se determination by GF AAS for digests of Sample A obtained after decomposition by conductive heating (blue ●) and MWAD (red ●). Vertical bars represent the standard deviation for*n* = 3.

As shown in Figure 7, there is a pronounced increase in the background signal with higher nitric acid concentrations. In addition, a decrease in the analyte signal was observed as the concentration of nitric acid increased, probably due to diffusion effects induced by convection currents (Figure S2). Therefore, for selenium quantification in the digests, dilution was applied to achieve a final nitric acid concentration of 1%. Under these conditions, the low RA contributed to prolonging the graphite tube lifetime, allowing a total of 1036 atomization cycles in the GF AAS (Figure S3). The dilution step is necessary to prevent the degradation of the atomizer and also to avoid the matrix effects causing analyte signal suppression.

To verify matrix effects, two calibration curves were prepared with concentrations ranging from 20 to 120 μg L^–1^ (0.3 to 1.8 ng), in aqueous solution and in Sample A digestates diluted five times, obtained by MWAD. The calibration curves obtained exhibited similar slopes when the samples were diluted at least five times (0.00132 for the standard solution and 0.00139 for the standard addition method), both with correlation coefficients (*R* ^2^) above 0.999 (Figure S4). Although the sensitivity was approximately 5% higher in the presence of the sample matrix, this increase was not statistically different (95% confidence level, *t* test), indicating that the sample matrix did not considerably interfere with the analyte atomization. Considering that the standard addition calibration is more time-consuming, the external calibration method was selected to optimize analytical throughput.

The characteristic mass obtained for the standard Se solution for the digestates obtained by the conductive heating methods, MWAD and MIC were 44.5, 44.1, and 44.2 pg, respectively, with no statistical difference (95% confidence level, ANOVA) among the values.

### 3.5 Comparison of Sample Preparation Methods

The limits of quantification (LOQ) and detection (LOD) were determined based on ten consecutive measurements of the analytical blank, following IUPAC recommendations.^33^ For each sample preparation method, the corresponding reagent solution was used as the blank: 5 mol L^–1^ nitric acid with 2 mL of 30% w/w hydrogen peroxide for the conductive heating; 2.5 mol L^–1^ nitric acid with 3 mL of 30% w/w hydrogen peroxide for MWAD; and 0.25 mol L^–1^ nitric acid with 300 mg of microcrystalline cellulose for MIC. The digestates obtained from the conductive heating and MWAD were diluted 7 and 5 times, respectively, for analysis by GF AAS. The LOD and LOQ values are shown in Table 2.

**Table 2:** LOQ and LOD of Optimized Methods for the Decomposition of Dietary Supplements

Parameters	Conductive heating	MWAD	MIC
Nitric acid, mol L^–1^	5.0	2.5	0.25
Maximum sample mass, mg	250	350	200
LOD, μg g^–1^	1.71	0.84	0.08
LOQ, μg g^–1^	5.66	2.37	0.21

The LOQ obtained using the MIC was approximately 100 times lower than that obtained with the conductive heating. Although the sample masses used were similar, the greater dilution required for conductive heating (7.5×) explains the substantial difference in LOD and LOQ values. Similarly, the approximately 5 times dilution required for MWAD also negatively affected the detection limits, even though a larger sample mass was used.

Table 3 presents a comparison of the LODs and LOQs of the proposed work with those presented in the literature.

**Table 3:** Conditions of Sample Preparation and LODs Reported in This Work and in the Literature for Determination of Selenium in Dietary Supplements

Digestion method	Sample preparation	Technique	LOD, μg g^–1^	LOQ, μg g^–1^	refs
Conductive heating	250 mg of sample, 5 mol L^–1^ HNO_3_ and 2 mL of 30% w/w H_2_O_2_	GF AAS	1.71	5.66	This work
	Heating program for 5 h and a maximum temperature of 160 °C				
MWAD	350 mg of sample, 2.5 mol L^–1^ HNO_3_ and 3 mL of 30% w/w H_2_O_2_		0.84	2.37	This work
	Heating program for 60 min and maximum temperature of 180 °C at 1260 W				
MIC	200 mg of sample, with 300 mg of microcrystalline cellulose, in pellets, and 0.25 mol L^–1^ HNO_3_ as absorbing solution		0.08	0.21	This work
	Heating program: (i) 900 W for 5 min and (ii) 0 W for 15 min (cooling step)				
Conductive heating	10 mL of concentrated HNO_3_ and 0.05 mL of concentrated HF	ICP-SFMS	0.05	not informed	^25^
	Overnight predigestion followed by a heating program at 110 °C for 120 min				
MWAD	250 mg of sample, 4 mL concentrated HNO_3_, 1.5 mL concentrated HF and 1 mL of 30% w/w H_2_O_2_	HR-CS GF AAS	0.04	not informed	^23^
	Heating program at 20 min at 300 W				
MWAD	200 mg of sample. Nine mL concentrated HNO_3_ and 1 mL of 30% w/w H_2_O_2_	HR-CS GF AAS	0.10	0.34	^24^
	Heating program for 56 min and maximum temperature 190 °C				

Comparing the LODs obtained in this study with those previously reported in the literature, the value achieved for selenium (0.08 μg g^–1^) using the MIC method was comparable to that reported by Augustsson et al.^25^ (0.05 μg g^–1^) for dietary supplements analyzed by ICP-SFMS, and Krawczyk^23^ (0.04 μg g^–1^) and Almeida et al.^24^ (0.10 μg g^–1^), both using HR-CS ETAAS. However, in both of those studies, concentrated acids were employed for sample digestion, whereas in the present work, similar sensitivity was achieved using a nitric acid solution that was up to 50 times more diluted. Additionally, it is important to mention that it was not found in the literature data, studied using the MIC method for the digestion of this type of sample for further Se determination.

The optimized sample preparation methods proved to be effective for the decomposition of dietary supplements. The MIC demonstrated superior decomposition efficiency, with reduced reagent consumption and waste generation, as well as low levels of carbon and acidity in the digestates, an important advantage for techniques such as ICP-MS,^27,33^ cold vapor generation coupled to microwave-induced plasma optical emission spectrometry (CVG-MIP-OES),^30^ IC,^29^ ion-selective electrode (ISE),^44^ and others.^35^

Wet digestion methods are widely employed for the decomposition of dietary supplement samples due to their greater availability, ease of operation, and lower operational costs. Although they provide lower decomposition efficiency compared to MIC, MWAD, for instance, stands out by the efficient energy transfer promoted by microwave heating, which favors sample decomposition under milder conditions, thereby reducing reagent consumption and chemical waste generation.^14,45^ In contrast, conductive heating digestion is limited by longer preparation times, the requirement for more concentrated acids, and lower mineralization efficiency.^16,17^ Nevertheless, this method can still provide satisfactory results for less complex matrices or laboratories with limited access to advanced instrumentation.

The greenness of the sample preparation methods was evaluated using the AGREEprep software, which scores ten of the GSP criteria from 0 to 1, where higher values indicate greener procedures.^46^ The input data are described in Table S2.

All methods were conducted ex situ, which reduced the overall score due to additional handling steps and the need for a specific laboratory infrastructure. The use of nitric acid, a flammable, corrosive, and toxic reagent, also negatively impacted the assessment of safer reagents, although the use of diluted solutions and small reagent volumes helped mitigate this effect.

Regarding the sample economy, MWAD used the largest sample mass, followed by conductive heating and MIC, which favored MWAD in this aspect. The sustainability of materials received intermediate scores for all methods, since the digestion vessels, though nonrenewable, can be reused after proper decontamination.

Waste generation was similar among the methods, with each producing approximately 25 mL of digestate. However, differences were more evident in sample throughput: MIC showed the best performance (24 samples h^–1^), followed by MWAD (12 samples h^–1^) and conductive heating (2.4 samples h^–1^), consistent with the shorter processing time of combustion-based digestion.

In terms of automation, the three methods were comparable, as all involved semiautomated steps. Energy consumption revealed a stronger contrast: conductive heating showed the lowest greenness due to the higher power demand per sample; MWAD presented intermediate energy use, and MIC achieved the best performance owing to its lower power requirements and faster digestion.

For postsample configuration, all methods employed GF AAS, which is penalized in the AGREEprep framework due to its high operating temperature, energy demand, and limited throughput. Operator safety was influenced by the number of hazard pictograms associated with the reagents: five for the wet digestion methods (corrosive, toxic, oxidizing, and flammable) and six for the MIC, given the additional use of pressurized oxygen.

Overall, the MIC achieved the highest AGREEprep score (0.49), driven by its higher throughput and lower energy demand, followed by MWAD (0.31) and conductive heating (0.20). Default weighting factors were used for all calculations. The AGREPREP evaluation scores on the proposed methods are shown in Figure 8.

**Figure 8:**
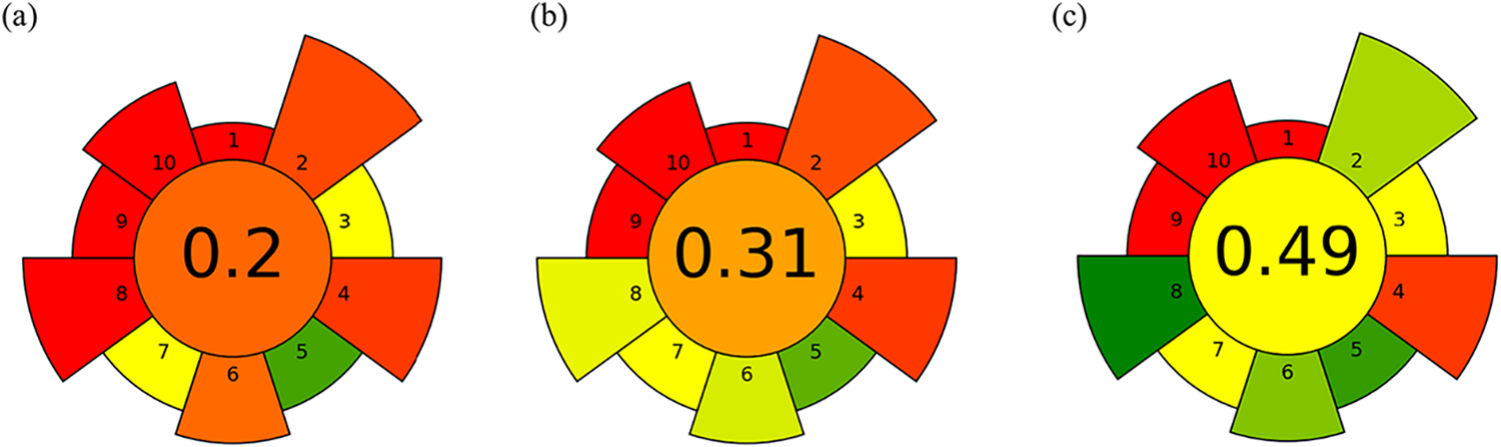
Greenness evaluation of the proposed methods using AGREEprep metrics: (a) conductive heating, (b) MWAD, and (c) MIC.

The accuracy of the proposed methods was evaluated from the total selenium concentrations obtained. The results showed no significant difference among them (95% confidence level, ANOVA test), indicating agreement between the methods. The selenium concentration indicated on the label of Sample A was 30 μg g^–1^. The measured values were 33.51 μg g^–1^ for conductive heating, 32.13 μg g^–1^ for MWAD, and 31.48 μg g^–1^ for MIC, all consistent with the values declared by the manufacturer.

Additionally, accuracy was evaluated by the analysis of CRM SELM-1 using the same decomposition methods. The results showed selenium concentrations that were equivalent to the certified value (95% confidence level, *t* test), with agreement ranging from 96.7 to 99.5%. These findings demonstrate the accuracy of the developed methods and indicate that both, from simpler equipment to a more advanced method, are suitable for the decomposition of dietary supplement samples, even when using diluted nitric acid. Although the MIC system requires a higher initial investment due to the type of vessels (quartz), its operational cost per analysis is considerably lower due to the reduced consumption of diluted nitric acid (approximately 0.11 mL of HNO_3_ 65% w/w, high purity) and shorter digestion time. Moreover, the lower residual acidity of MIC digests contributes to extending the tube lifetime and further reducing operational costs. Additionally, when the choice of a green procedure is a point of consideration, MIC proved to be a more green method considering the low acid consumption, analytical throughput also providing quantitative results, and agreement when a CRM was used.

### 3.6 Quantification of Total Selenium in Dietary Supplement Samples

Selenium was successfully quantified in dietary supplement samples using all three evaluated decomposition methods and the results were provided in Table 4.

**Table 4:** Se Concentration in Dietary Supplements was Calculated According to Different Sample Decomposition Methods. Results are Expressed as the Mean ± Standard Deviation for the Conductive Heating and MWAD (*n* = 3) and MIC (*n* = 4)

Sample	Se, μg g^–1^			
	conductive heating	MWAD	MIC	Se content^a^
A	33.51 ± 1.72	32.13 ± 0.55	31.48 ± 0.66	30
B	36.42 ± 1.44	33.91 ± 1.23	36.14 ± 1.75	34
C	132.3 ± 6.3	129.8 ± 3.5	124.9 ± 5.1	132
D	211.5 ± 7.2	218.1 ± 3.8	218.8 ± 8.8	200

a Reported on the dietary supplement label.

For Samples A, B, C, and D, selenium concentrations varied from 31.48 to 33.51, 33.91 to 36.42, 124.9 to 132.3, and 211.5 to 218.8 μg g^–1^, respectively. No statistical differences were observed among the selenium concentrations determined by the three decomposition methods (95% confidence level, ANOVA test) for each sample. Furthermore, the selenium concentrations were in agreement with the values declared on the product labels (95 to 111%).

The apparent differences in the RSD values observed for samples C and D for the MIC method could be associated with the combustion step of these samples, since the analyte recovery depends on the combustion and the subsequent step of reflux. However, the RSD values are relatively low (about 4%), and no statistical difference was observed among the results (95% confidence level, ANOVA) for all sample preparation methods investigated. For all samples, the relative standard deviations were below 5%, complying with the AOAC^47^ repeatability acceptance criteria for the corresponding analyte concentration level, which confirms the reliability and precision of the analytical procedure.

Although only four samples were analyzed, they encompass the main categories of selenium-containing dietary supplements currently marketed, representing formulations based on minerals or vitamins, probiotics, polysaccharides, and selenium-enriched yeast. Therefore, the results obtained are considered representative of the composition and selenium levels typically found in commercial dietary supplements.

According to ANVISA, the minimum and maximum RDI values of selenium for adults are set at 8.2 μg and 319 μg, respectively.^10^ These values are based on current dietary intake recommendations from international authorities such as the FDA^6,7^ and EFSA.^8,9^ In general, across the five dietary supplement samples analyzed, selenium content ranged from 92.6 to 129% of the declared value and remained below the maximum RDI. This corresponds to approximately 10, 11, 40, 67, and 4% of the maximum daily intake for Samples A, B, C, and D, respectively. Therefore, all samples comply with the specifications of the regulatory agency, ensuring that they are safe for daily consumption at the recommended doses stated on their respective labels.

## Conclusions

This study demonstrated the applicability and effectiveness of three sample preparation methods for dietary supplements aimed at selenium quantification by GF AAS, with a focus on sample preparation. The comparative evaluation of the methods: conductive heating, MWAD, and MIC, allowed for the identification of the advantages and limitations of each approach. It is important to highlight that the evaluated methods were compatible with the use of diluted nitric acid, which represents a significant reduction in reagent consumption and chemical waste generation, aligning with the principles of Green Chemistry. Moreover, the use of digests with a lower RA contributed to extending the operational lifetime of the graphite tube and reducing operational costs. The overall environmental performance of the methods was further assessed using the AGREEprep metric, which quantitatively confirmed the superior greenness of the MIC approach; this MIC method provided lower LOQ and LOD, even though smaller sample masses were used. The selenium concentrations in all analyzed samples were in accordance with the specifications established by ANVISA, which set a daily intake limit of 319 μg for adults.

## Supplementary Material



## Notes

Information about selenium content and composition of the dietary supplements analyzed (Table S1), a summary of sample preparation decomposition procedures evaluated using the AGREEprep software (Table S2), absorbance signal profiles for Se and background in digests obtained by the sample preparation methods investigated (Figures S1 and S2), a comparison between the new graphite tube and the graphite tube used in this work (Figure S3), and calibration curves obtained by external calibration and standard addition by GF AAS (Figure S4) (PDF)
