# Supplementary material for: Investigation of Sample Preparation Methods of Dietary Supplements for Total Selenium Determination
Source: ACS Omega. 2025 Dec 4;10(49):60752–62. doi: 10.1021/acsomega.5c08938 (PMC12713496; doi:10.1021/acsomega.5c08938)
Supplement: Supplementary file 1 [file ao5c08938_si_001.pdf]

Investigation of Sample Preparation Methods of Dietary Supplements for Total Selenium Determination

Larissa M. A. Oliveira, Diogo P. Moraes, Juliana S. F. Pereira*

Rio Grande do Sul Federal University, Chemistry Institute, Department of Inorganic Chemistry, Porto Alegre-RS, 91501-970, Brazil

* Corresponding author

Juliana S. F. Pereira

Email: julianasfp@ufrgs.br

https://orcid.org/0000-0002-1752-620

Supporting Information

Table S1. Selenium content, dosage form and ingredient composition reported on the labels of dietary supplements used in this study.

Sample code	Se content, µg	Tablet, mg	Origin	Ingredient composition
A	30	1050	EUA	*Lactobacillus acidophilus*, vitamins A, B1, B2, B6, B9, B12, C, D and E, niacin, pantothenic acid, biotin, Ca, P, Mg, Fe, Zn, Mn, Se and I.
B	34	640	Brazil	Vitamins A, B1, B2, B3, B5, B6, B12, C, D and E, folic acid, Fe, Mn, Mg, Se and Zn.
C	132	1300	Brazil	Beta-glucan from yeast *Saccharomyces cerevisiae*, vitamins C and D, and Se.
D	200	300	EUA	Selenized *Saccharomyces cerevisiae* yeast, vegetable cellulose, vegetable stearic acid and vegetable magnesium stearate.

Table S2. Description of the sample decomposition procedures evaluated using the AGREEprep software in this work.

Criterion	Conductive heating	MWAD	MIC	Weight
1. Sample preparation placement	Ex situ	Ex situ	Ex situ	1
2. Hazardous materials	1.8 mL 65% w/w HNO_3<br>2 mL 30% w/w H_2O_2	1.5 mL 65% w/w HNO_3<br>3 mL 30% w/w H_2O_2	0.1 mL 65% w/w HNO_3	5
3. Sustainability and renewability of materials	Materials are not sustainable or renewable but are used several times	Materials are not sustainable or renewable but are used several times	Materials are not sustainable or renewable but are used several times	2
4. Waste	25 mL	25 mL	25 mL	4
5. Size economy of the sample	0.25 g	0.35 g	0.20 g	2
6. Sample throughput	2.4 samples per hour	12 samples per hour	24 samples per hour	3
7. Integration and automation	1 step (digestion), semi-automated system	1 step (digestion), semi-automated system	1 step (combustion), semi-automated system	2
8. Energy consumption	779.2 W h^{-1} by sample<br>12 positions	58.3 W h^{-1} by sample<br>12 positions	9.4 W h^{-1} by sample<br>8 positions	4
9. Post-sample preparation configuration for analysis	G FAAS	G FAAS	G FAAS	2
10. Operator's safety	5 hazards	5 hazards	6 hazards	3

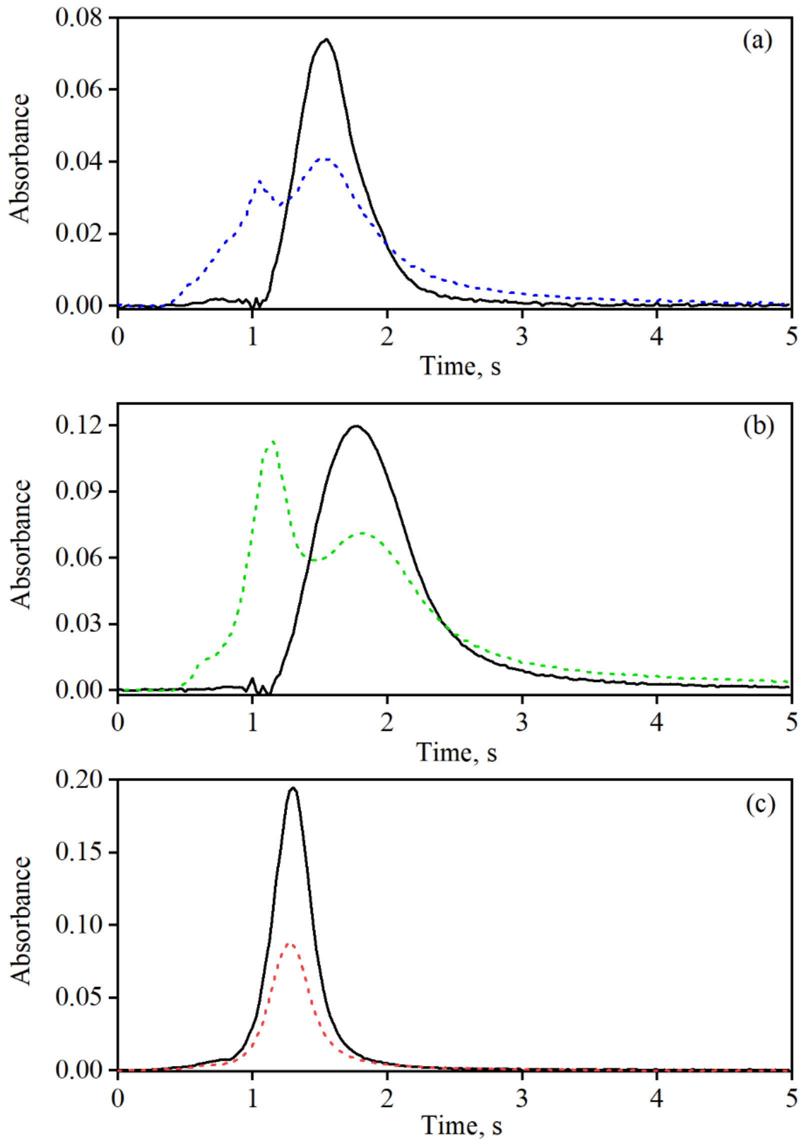

Figure S1. Analyte (—) and background (---) in digests of Sample A decomposed by conductive heating (a), MWAD (b), and MIC (c). The pyrolysis and atomization temperatures were 1200 °C and 2000 °C, respectively.

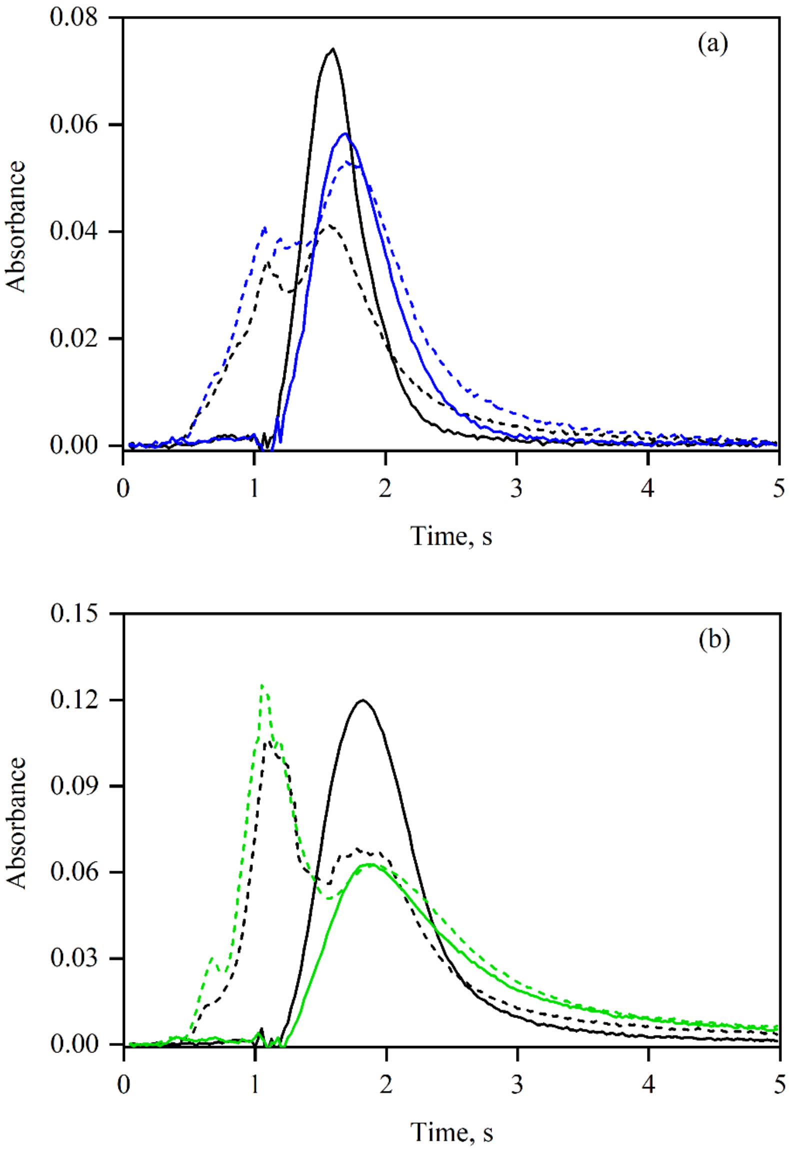

Figure S2. Analyte (—) and background (---) signals obtained in selenium determination by GF AAS for digestates from: (a) conductive heating with 1.0% (black) and 7.5% (blue) HNO_3, and (b) MWAD with 1.0% (black) and 5.0% (green) HNO_3.

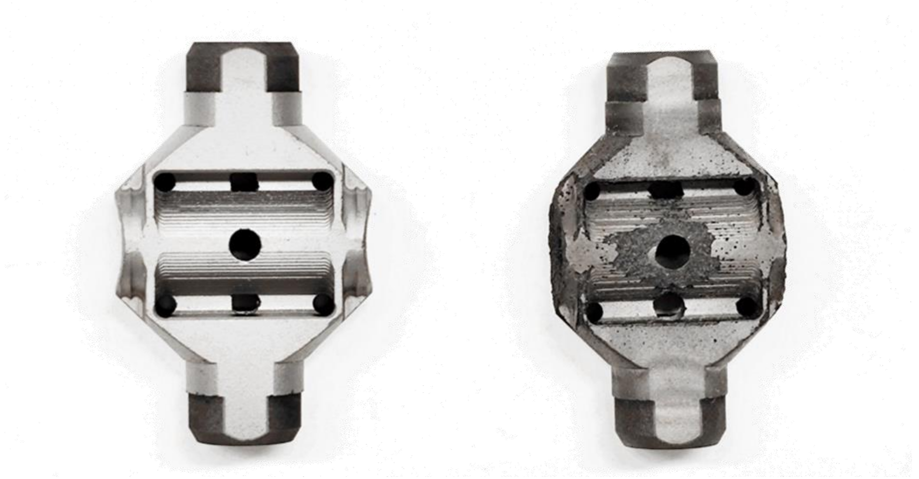

Figure S3. Comparison between the new graphite tube (left) and the graphite tube used in this work (right) after 1036 firings conducted during the experiments.

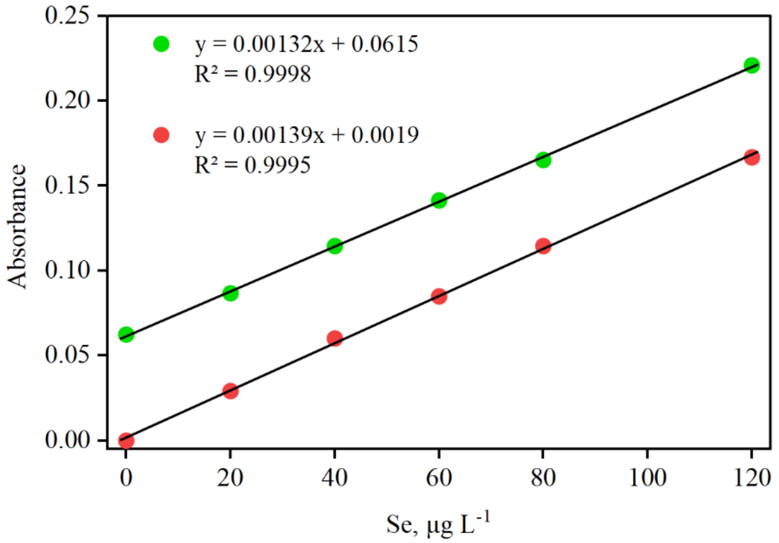

Figure S4. Calibration curves obtained by external calibration (●) and standard addition (●) by GFAAS.
